# Editors’ Book Table

**Published:** 1873-03

**Authors:** 


### Editors' Book Table.

[NOTE.—All works reviewed in the columns of the CHICAGO MEDICAL JOURNAL may be found in the extensive stock of W. B. KEEN, COOKE & CO., whose catalogue of Medical Books will be sent to any address upon request.]

BOOKS RECEIVED.

*Dental Caries and its Causes.* An Investigation into the Influence of Fungi in the Destruction of the Teeth. By DRS. LEBER and ROTTENSTEIN. Translated by THOMAS H. CHANDLER, D.M.D., Professor of Mechanical Dentistry in the Dental School of Harvard University. With Illustrations. Philadelphia: Lindsay & Blakiston. 1873. 8vo. Pp. 103. Extra Cloth, $1.50.

The best exposition of the subject, it has been our fortune to meet. Dr. Chandler has merited the thanks of both dentists and physicians, in furnishing them this *brochure* in English dress.

*First Annual Report of the Supervising Surgeon of the Marine Hospital Service of the United States. For the Year* 1872. Containing a Brief Historical Sketch of the Service, from the date of its Organization in 1798.

Our thanks are due John M. Woodworth, M.D., the Supervising Surgeon, for a copy of his report. At the time of his appointment, we congratulated the Department on securing his services, and the present report amply sustains our opinion. We are particularly pleased at seeing under the caption, "Proposed New Hospitals," this sentence italicized: "*I particularly favor constructing all the hospitals of wood, and destroying them after ten or fifteen years, both as a sanitary and economical measure, and building new ones in their stead.* The prime object to be attained, is to secure favorable results in the treatment of diseases and injuries—an object which has been, in the past, subordinated to architectural design, and frequently to the favoring of certain localities. A permanent building of brick or stone should be constructed adjacent to, and separate from, each hospital building, to contain the heating apparatus and laundry." This is the true, the sound and modern doctrine, and the city of Chicago (or Cook Co.) should thoroughly investigate it, before entering upon the business of reconstructing its general hospital. Meanwhile, we extend our thanks to Surgeon Woodworth for his frank statement.

*The Medical and Surgical History of the War of the Rebellion,* (1861–65). Prepared in Accordance with the Acts of Congress, under the direction of Surgeon General JOSEPH K. BARNES, U.S.A. Washington: Government Printing Office. 1870. Part First, Medical Volume, pp. 726, and Appendix, pp. 365. Part First, Surgical Volume, pp. 650, Text; pp. xiv, Index, etc.

We have no time at present to write more than our thanks to the Surgeon General for these superb volumes, which demonstrate that in the hands of the Medical Department of the U.S.A., under his guidance, medical and surgical science will receive an impetus to be felt everywhere throughout Christendom. For our science can never be perfected by microscopic observation alone. The macrocosm as well as the microcosm is to be put under surveillance. Since the world began, there has never been a series of observations so comprehensive, and yet so minute and accurate, as those which are now first appearing in systematic form in these volumes. We shall look for each succeeding issue with high expectations.

*The Science and Art of Surgery.* Being a Treatise on Surgical Injuries, Diseases, and Operations. By JOHN ERIC ERICHSEN, Senior Surgeon to University College Hospital, and Holme Professor of Clinical Surgery in University College, London. A New Edition, Enlarged and carefully Revised by the Author. Illustrated by upwards of Seven Hundred Engravings on Wood. Vol. 1, pp. 781; Vol. 2, pp. 918.

It is superfluous to repeat the encomiums with which we have from time to time greeted the appearance of successive editions of this invaluable work. It stands pre-eminent among all the standard books on surgery. It is the great manual of practice. An old and well-thumbed copy of the American edition of 1860, stained and discolored by use and exposure, picked up, April 7, 1862, on the battle field of Pittsburg Landing, is at this moment on our office table, and we can well imagine the discomfiture of the late Confederate surgeon at its loss. On comparing the two copies, we find that each mirrors the practice of surgery at the time of issue. All through the volumes, we find evidence of most industrious and conscientious revision. Although much that had become obsolete is omitted, nevertheless, necessary additions have swelled the number of pages from 996 in our Confederate copy to

1699 in that before us. That copy has 417 illustrations, and this over 700. Hence the necessity of furnishing it in two volumes instead of one. The present edition is a splendid specimen of the printer's and binder's art, and the publisher cannot be too highly commended, for his good taste and liberality in sending out this cyclopædic treatise in such elegant garb. By the by, in turning over the leaves of our "Confederate copy" we happen on this prescription, hastily written in pencil on a scrap of paper, which shows several things needing no comment. We sincerely trust that the former owner of this book is now gathering, plentifully, of lawful greenbacks in the Sunny South, and not mourning for the enormous fees formerly paid him in Confederate scrip. Here is the prescription:

"Dr. Eve's Recipe for Gleet and chronic irritation of the urethra:

| | |
|---|---|
| Balsam Copaiva, . . . . . . . . . | 1 oz. |
| Pulv. Cubebs, . . . . . . . . . . | ½ " |
| P. Gum Arabic, . . . . . . . . . | 1 " |
| Spts. Nitre, . . . . . . . . . . | 1 " |
| Syrup Buchu, . . . . . . . . . . | 4 " |
| Cinnamon Water to fill black bottle. | |

Dose, 2 tablespoonfuls twice a day."

☞ Quite a number of Book Notices, already in type, are unavoidably crowded out of this issue, which will appear next month.

